# Multimodal Imaging and Lighting Bias Correction for Improved μPAD-based Water Quality Monitoring via Smartphones

**DOI:** 10.1038/srep27529

**Published:** 2016-06-10

**Authors:** Katherine E. McCracken, Scott V. Angus, Kelly A. Reynolds, Jeong-Yeol Yoon

**Affiliations:** 1 Department of Agricultural & Biosystems Engineering, The University of Arizona, Tucson, Arizona 85721, United States; 2 Mel and Enid Zuckerman College of Public Health, The University of Arizona, Tucson, Arizona 85721, United States

## Abstract

Smartphone image-based sensing of microfluidic paper analytical devices (μPADs) offers low-cost and mobile evaluation of water quality. However, consistent quantification is a challenge due to variable environmental, paper, and lighting conditions, especially across large multi-target μPADs. Compensations must be made for variations between images to achieve reproducible results without a separate lighting enclosure. We thus developed a simple method using triple-reference point normalization and a fast-Fourier transform (FFT)-based pre-processing scheme to quantify consistent reflected light intensity signals under variable lighting and channel conditions. This technique was evaluated using various light sources, lighting angles, imaging backgrounds, and imaging heights. Further testing evaluated its handle of absorbance, quenching, and relative scattering intensity measurements from assays detecting four water contaminants – Cr(VI), total chlorine, caffeine, and *E. coli* K12 – at similar wavelengths using the green channel of RGB images. Between assays, this algorithm reduced error from μPAD surface inconsistencies and cross-image lighting gradients. Although the algorithm could not completely remove the anomalies arising from point shadows within channels or some non-uniform background reflections, it still afforded order-of-magnitude quantification and stable assay specificity under these conditions, offering one route toward improving smartphone quantification of μPAD assays for in-field water quality monitoring.

Amid the ever growing concerns for safe drinking water around the globe, it has become increasingly important to develop a portable sensing platform that can report data on water quality in near real-time^1^,^2^,^3^. Towards this end, smartphones have been demonstrated as ideal, low-cost signal acquisition and reporting tools that have become increasingly available to all, with an estimated 3.4 billion smartphone subscriptions worldwide as of 2015^4^,^5^,^6^. Already smartphones have been demonstrated for optical sensing of electrochemical, colorimetric, and fluorescence-based assays, as well as for portable microscopy^7^,^8^,^9^,^10^,^11^,^12^,^13^,^14^. By taking advantage of the external forces driving both the widespread use of smartphones as well as the rapid improvements in their on-board cameras and processors, these devices emerge as potentially sensitive and programmable utensils for handheld data collection, analysis, and rapid sharing^5^,^15^,^16^.

As a sensing platform, microfluidic paper analytical devices (μPADs; essentially microfluidic devices fabricated on paper substrates) have also emerged separately as a favorably portable and low-cost means for detecting customizable classes of waterborne contaminants^17^. Building on the fundamental porous strip design of lateral flow assays (LFAs), μPADs can be fabricated using a simple cellulosic substrate and photolithographic processes to create hydrophilic flow channels that are prepared with user-selected target-responsive agents and which allow sample filtration from particulate debris as the sample flows through the paper fibers via capillary action^17^,^18^.

These designs have previously been used for detection of contaminants including viruses, fungi, heavy metals, and organic molecules, such as antibiotics and hormones, with useable sensitivity^19^,^20^,^21^,^22^,^23^,^24^. Through image analysis and measurement of wavelength-specific intensities from a colorimetric or scattering-based μPAD assay, smartphones can help to improve mobile sensing quality without sacrificing economy and portability^16^,^25^,^26^. Fluctuating and uncontrollable field conditions such as lighting, temperature, humidity, and paper surface uniformity, however, plague the consistent quality of reagent-based μPAD assays, even for comparison with standard curves run simultaneously^17^,^26^. Spatial variations in ambient lighting and lighting exposure may be minimized through the use of a secondary smartphone attachment or a lighting-control enclosure when imaging. However, reliance upon such attachments potentially limits a detection technique to one device model and does not evolve immediately with improving technology.

In this work, we investigated the application of a simple smartphone imaging and bias-correction processing technique to combat these uncertainties within μPAD sensing. Toward these ends, we developed and tested a triple-reference point normalization concept following FFT-based image pre-processing using digital bandpass filtration techniques (Fig. 1a–c). The benefits and limitations of this technique were assessed by its performance in handling changes in the lighting field (lighting angle ϕ; lighting source/quality) and in the imaging field (height of light source; background absorbance/reflectance characteristics) when comparing an array of channels under identical assay conditions. Further assessments of this technique were made by comparing the limits of detection and distinction between signals for multiple colorimetric- and fluorescence-based assays (detected at 90° from the channel surface) and a Mie scattering-based assay (detected at 65°) under varying lighting conditions (Fig. 1d). These assays were selected due to their similarity in detection wavelength (512–540 nm), such that we assessed detection from only one (green) 8-bit RGB channel from each smartphone image.

The assays also represented a range of targets with diverse optical contrast levels, from high contrast colorimetric changes that are visible with the naked eye to low contrast Mie-scattering which is visually imperceptible (Fig. 1e). Additionally, each assay – evaluating the interactions between diphenylcarbazide (DPC) and Cr(VI), N,N-diethyl-p-phenylenediamine (DPD) and total chlorine (TC), 8-hydroxypyrene-1,3,6-trisulfonic acid (HPTS) and caffeine, as well as IgG-microparticle immunoagglutination with *Escherichia coli* K12 – has been previously demonstrated by laboratory-based spectroscopic quantification using precisely controlled lighting^27^,^28^,^29^,^30^. Thus, these targets allowed comparison between our smartphone algorithm and established levels of detection. This variety of targets, optical quantification methods, and levels of contrast afford a simultaneous evaluation of the broad applicability, sensitivity, and specificity of smartphones used as multi-target optical analytical devices for mobile water quality monitoring.

## Results and Discussion

### Triple-Reference Point Normalization Algorithm for μPAD Reflected Intensity

**Figure 1:**
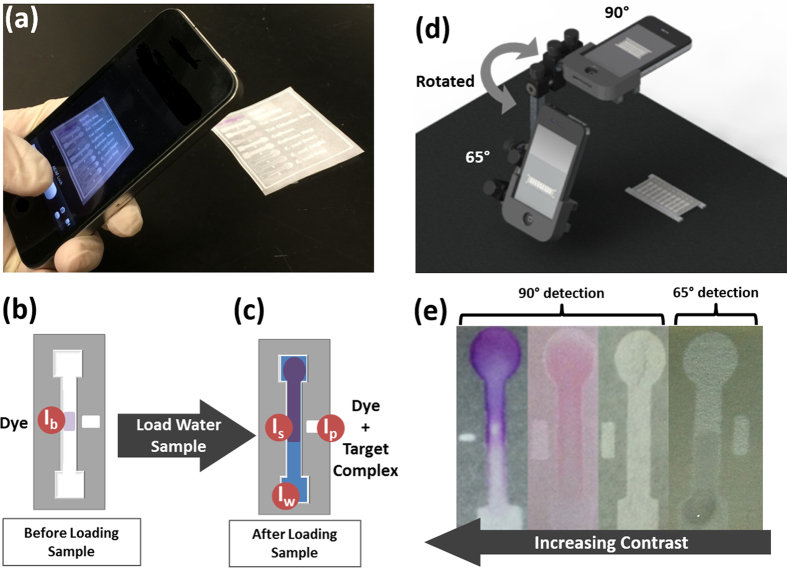
(**a**) Multi-target water quality analysis through paper-based smartphone detection. (**b**) Hydrophilic channels are patterned in cellulose filter paper using SU-8 (negative) photoresist. After fabrication, channels are pre-loaded with target-sensitive reagents, providing a reference reflected intensity for the paper-surface background (I_b_). (**c**) After a water sample is loaded, it flows along the length of the channel. If the water sample contains the reagent-specific target, an optically-detectable change in the channel’s reflected intensity is produced (I_s_). Two additional reference intensity values are also available: the peak reflected intensity (I_p_), or “pure white” reference, indicating the maximum incident light available to the paper strip; and the wetted paper reflected intensity (I_w_), indicating the change in reflected intensity that is attributable to only the saturation level of the cellulose channel. (**d,e**) Targets are identified and quantified optically through smartphone image analysis of each channel at empirically determined detection angles (90° for absorbance and fluorescence detection, and 65° for Mie scattering detection).

Prior to each experiment, the smartphone (iPhone 4S; Apple, Cupertino, CA, USA) was fastened into a precisely controlled angular rotation stage and the μPAD was fixed within the imaging window at a defined angle relative to the smartphone camera lens. The auto-exposure and auto-focus were locked on the central paper channel surface at 90° for Cr(VI), TC, and caffeine assays, and at 65° for *E. coli* K12 assays – the optimum scattering detection angle determined from previous work^30^ (Fig. 1d).

Two smartphone images were collected for each μPAD assay: the first image taken after loading and drying 2 μL of the target-specific reagent solution in the center of the channel, and the second image taken after loading 8 μL of the target water sample to the channel inlet and allowing it to flow completely through the channel. From these two images, four average reflected intensity values were measured for each μPAD channel from the 8-bit green channel of the RGB smartphone image: one target-reagent signal point (I_s_) and three reference points (I_b_, I_p_, I_w_) taken to facilitate robust detection under uncontrolled experimental conditions of light, humidity, and channel surface uniformity (Fig. 1b,c).

In the first image, the mean intensity of the channel center was measured where the reagents were loaded and dried, and served as a background intensity (I_b_) reference for the native coloration of the paper channel surface and target-sensitive agent (Fig. 1b).

In the second image, taken after the target water solution flowed through the channel, the mean intensity of the channel center point was measured and provided the target-reagent signal intensity (I_s_) (Fig. 1c). Two additional reference points were measured from the same image. A peak white reference (I_p_) point was taken from the mean intensity of a dry rectangle patterned adjacent to but separated from the μPAD channel. This identified the maximum illumination available to the dry paper, serving as a reference for the unique illumination of each channel center and accounting for the original quality of the paper as well as changes made to the paper during the fabrication process. A wetted reference (I_w_) point was also taken at the sample loading inlet, where the paper channel was saturated with the target water sample but not with the target-specific reagent. This allowed calibration for moisture-induced coloration differences between channels, which varies based on the effects of humidity and fabrication of separate channels.

The following normalization scheme was then developed and selected empirically by its capacity for inter-channel comparison, as discussed in the following section. The wetted target-reagent signal was normalized as I_s_/I_w_, which accounts for the target-reagent signal change as a fraction of the signal change due to moisture in the channel. The dry reference was normalized as I_b_/I_p_, which accounts for the maximum illumination available to the reagent in the dry channel as a fraction of the maximum total illumination available to the dry paper within the given lighting conditions. As such, this dry reference serves as an efficiency term indicating the percent of the maximum available light intensity that measurably changed as a result of target-reagent complex as opposed to the native coloration of the reagent on paper. The target-reagent green-channel pixel intensity (I) normalized to these three reference points can thus be defined as:


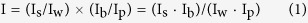


Similarly, the triple-reference normalized intensity for a deionized (DI) water control (I_0_) in the presence of the same reagent in the μPAD can be defined as:


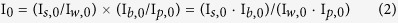


Using these normalized intensities, the following relative optical measurements can be defined as:


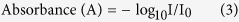



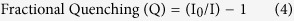



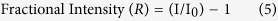


### Lighting Bias Error Characterization for Differential Illumination

**Figure 2:**
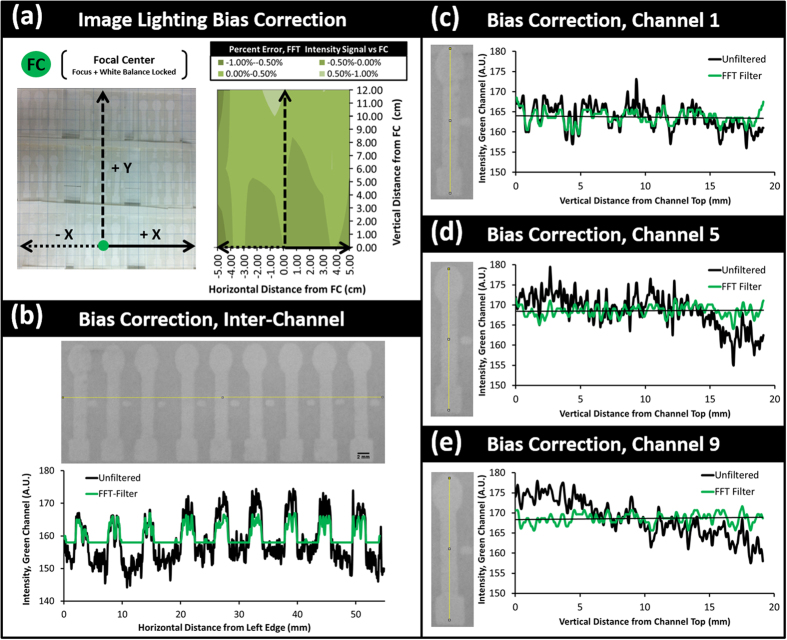
((**a**), left) Evaluation of the unfiltered lighting bias both horizontally and vertically across DI water control μPAD channels within a 12 cm × 12 cm viewing region. Each channel signal intensity (I_s_) and the three reference intensities (I_b_, I_p_, and I_w_) used within our mathematical normalization scheme were measured for each channel, and then were fractionally compared with the same measurement from the focal center channel. The maximum quantified error by normalization without initial image filtering was 12% ((**a**), right) To mitigate this lighting bias, a fast-Fourier transform (FFT) bandpass filter was applied to the original image, which reduced the error between channels and the focal center to under ± 1%. (**b**) This FFT bias correction alone allowed for a more uniform intensity signal measurements both between adjacent μPAD channels and (**c–e**) vertically within each single channel. This enabled the use of triple-reference point normalization within each channel as well as comparison between channels.

To assess the variation in only lighting conditions and paper surface uniformity between neighboring channels, a series of μPADs were positioned across an imaging region of 12 cm × 12 cm (Fig. 2a, left). These devices were imaged in the absence of a reagent both with and without a DI water target sample matrix to test initially for channel equivalence. The auto-focus and white balance were locked on the lowest central channel, which served as the focal center (FC) reference channel to which all other channels were compared. Under uniform lighting and paper surface conditions, the expected reflected intensities measured from each channel would be equal to the reference channel.

Under the observed environmental lighting conditions, however, anisotropic variation in reflected light intensity was identified across each dry μPAD channel surface, with lesser signal disparity horizontally between channels (ΔI_p,horizontal_ = 0.12 × I_p,avg_, i.e. the error is 12% of its average value, where Δ indicates percent error) (Fig. 2b) and greater disparity vertically along single channels (ΔI_p,vertical_ = 0.23 × I_p,avg_) (Fig. 2c–e). Between tests, this variation between the average incident green intensity value for each channel was also amplified by differences in paper surface characteristics even under similar lighting conditions (ΔI_p,trials_ = 0.15 × I_p,avg_). Under differing lighting conditions across the same imaging area between trials (fluorescent ambient light, λ_peak,green_ = 544 nm and smartphone LED flash lighting, λ_peak,blue_ = 445 nm), this lighting bias became more pronounced (ΔI_p,lighting_ = 0.18 × I_p,avg_). Thus, under the evaluated conditions, a similar test run under theoretically identical lighting conditions could result in smartphone-measured intensity values with combined error of ±50% from the average intensity for equivalent measurements, even before the introduction of error due to target-reagent assay variability (ΔI_p_ = 0.53 × I_p,avg_). With even slightly differing background lighting conditions, as is unavoidable under ambient lighting conditions and without a secondary lighting-control attachment, this strictly lighting-based error increases even further (ΔI_p_ = 0.68 × I_avg_).

Even following mathematical normalization of the DI water assays according to the triple-reference point algorithm, the combined lighting bias factors resulted in a standard error of ±8–21% relative to the reference signal taken at the focal center (FC) channel when measuring the absorbance (±8% error), quenching (±16%), and relative intensity (±21%) of the theoretically identical deionized water samples. These errors were measured between channels within the same image at distances of ±2–10 cm (p < 0.05), and are attributable to the gradated global lighting variation (or rather the camera’s characteristics in emphasizing the FC channel) affecting each channel differently along both its length and width according to its location within the imaging area (Fig. 2b–e).

### Lighting Bias Correction Using an FFT-based Digital Bandpass Filter

To compensate and correct for this lighting bias without a smartphone attachment, a fast-Fourier transform (FFT)-based digital bandpass filter was used^31^. Following the translation of the original channel image into the frequency domain, image aberrations outside of a selected feature size window (pixels: 3 ≤ n ≤ 30) were smoothed through the subtraction of a Gaussian blurred replicate of the same feature. In the subsequent image transferred back to the spatial domain, global features – in this case, the lighting bias – were balanced and their obscuring effect was diminished. The adverse consequence of this image filtering technique, however, is that while it attenuates the lighting bias, it also truncates the full range of intensity values measured within the original image. Thus, while the variation in intensity from each end of the channel may be reduced, the magnitude of intensity change between the background reference and the signal of interest is also reduced by up to 12% under tested conditions (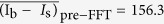 and 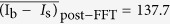 for assaying 50 ppm Cr(VI) using DPC).

FFT bandpass filtering and the empirically selected normalization scheme were used for the same images from the same lighting-bias control tests, enabling the lighting bias signal standard error to drop to ±1% relative to the focal center, with this maximum error experienced within 2 cm of the vertical image edge and within 1 cm of the horizontal image edge. Closer to the focal center, the lighting bias signal dropped to ±0.5%. The manifestation of this standard error averaged over the whole 12 cm × 12 cm imaging area for each measurement type – normalized absorbance, quenching, and relative intensity – was thus described as the assay illumination error (I.E.). The I.E. served as a threshold of detection below which the signal of the target-reagent complex is not distinguishable from the error due to the image illumination.

This processing and normalization mechanism was applied throughout the subsequent fitness tests, and later the single-target and multiplex target experiments, in order to permit comparative consistency between assays. Although the image processing was conducted off-board for this work, adaptation of the same FFT processing algorithm may be used within the smartphone to allow for the final and full adaptation of this concept for field-sensing applications.

### Fitness Testing of Combined Normalization and Filtration Technique

The combined effect of this triple-reference normalization algorithm with the FFT digital bandpass filtered images was evaluated for its performance in handling changes in the incident lighting angle (ϕ), the imaging height, the quality of the imaging background, and the quality of incident lighting. In each test, nine adjacent μPAD channels on a single μPAD were fixed within the smartphone imaging field and the white-balance and focus were locked on the detection region of the central channel (channel 5), which served as the new focal center (FC). The μPADs were imaged following the same process as used for the target-reagent assays, but again in the absence of a reagent and with only a deionized (DI) water target sample matrix. Their fractional intensity (R, defined in Eq. 5) was then compared with the FC as a measurement of error between channels. These fitness tests allowed better understanding of the appropriate conditions and potential limitations for the use of this technique for theoretically identical channels.

**Figure 3:**
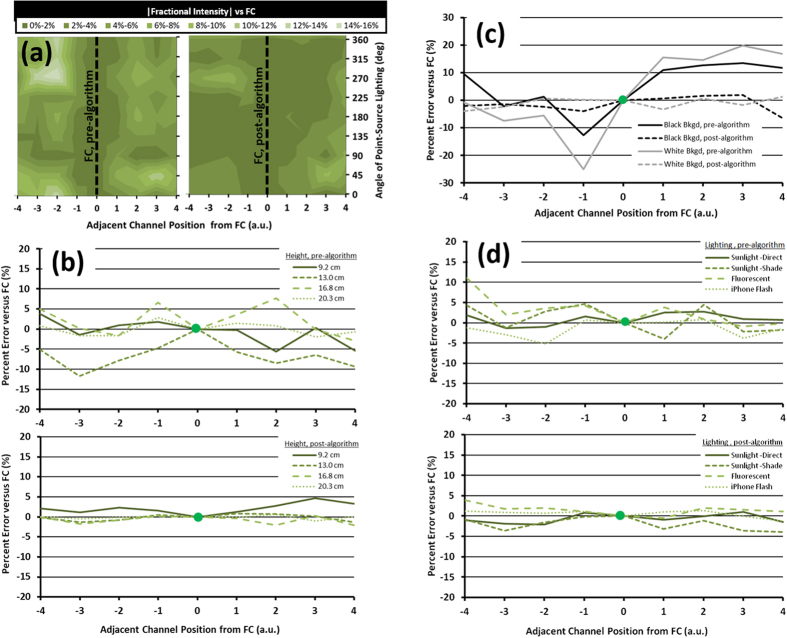
Fitness testing between a standard normalization method (I/I_0_) and the combined triple-reference normalization technique using FFT digital bandpass filtration of smartphone images. Adjacent channels within a nine channel array were imaged and compared with a focal center (FC) channel on which the image white balance and focus were locked. The normalized relative intensities and the standard error between testing methods were compared for (**a**) directional angles of point source lighting (45° increments between 0°–360°), for (**b**) increasing imaging height (9.2 cm–20.3 cm), for (**c**) different imaging backgrounds (black and white), and for (**d**) different qualities of lighting (natural direct and shaded sunlight, indoor fluorescent light, and smartphone flash light) (n = 8).

Incident lighting angle (Fig. 3a): As a test for the effects of directional lighting, the nine channel μPAD array was positioned horizontally and then revolved and imaged at ϕ = 45° intervals along a circumferential path (radius = 92 cm) around a fluorescent point light source. For each image, the orientation of the μPADs were maintained such that when ϕ = 0° the channels were illuminated from the direction of their inlet, and when ϕ = 180° they were illuminated from the direction of their outlet. This fixed μPAD orientation allowed for testing of the algorithm amid known differential illumination of each channel. First, each channel was analyzed using the original unfiltered images and their relative intensity signals were measured and compared with that of the FC channel (Fig. 3a, left), showing significant variance (up to 14%, p < 0.05) in fractional intensities. These error patterns may indicate that although error appears to generally increase with increasing distance from the reference point, as expected, this is not exclusively the case. Rather, for channels in closer proximity to a reference point (positions 2–3), error appears to be more associated with channel-specific conditions, potentially paper surface characteristics. In all locations error is also significantly associated with partial shading of individual channels (p < 0.05). After application of the algorithm, the error between each channel’s relative intensity measurement and that of the FC was greatly reduced (2–5% for most channels) (Fig. 3a, right). These error patterns match with the partial shading seen in the same images prior to FFT correction, suggesting that while the algorithm accounts adequately for general channel-specific lighting variation, it cannot fully account for extreme conditions such as partial shading.

Imaging height (Fig. 3b): Height-based error was tested by imaging the μPAD array under a constant diffuse fluorescent light source at increasing height (9.2 cm through 20.3 cm) and by comparing the inter-trial standard error of relative intensity measurements for DI water control assays. Before the algorithm, the errors in relative intensity compared to the FC were quite substantial (up to 12% at 13.0 cm) with inconsistent trends, while those at 20.3 cm were an exception (Fig. 3b, top). These patterns suggest that height changes cause or allow lighting errors to interfere with channel signal detection, potentially through white balance correction of the increasing background area. With the algorithm, this inter-channel variability was virtually eliminated for all heights, with the greatest variability seen only at the lowest position, where again partial shading reduces lighting of the channels (Fig. 3b, bottom).

Quality of background imaging (Fig. 3c): Addressing these concerns for the background’s contributions to white balance error and consequential signal noise, two monochromatic backgrounds – white and black – were tested. Under a diffuse fluorescent light and fixed height of light source, the white and black backgrounds both saw pronounced error between channels, with greater changes in relative intensity measured for white (maximum error = 25%) over black (maximum error = 13%) backgrounds. Both backgrounds demonstrated similar trends in error patterns, suggesting individual channel characteristics play a prominent role when single-image background variability is eliminated. With the algorithm, these errors were virtually eliminated (<3%), and there was no statistically significant difference between each channel (p < 0.05). Therefore, the algorithm appears to be able to manage individual channel irregularities, and fixed height and background characteristics may help to control full-image lighting variability that the algorithm cannot overcome.

Quality of incident lighting (Fig. 3d): Finally, the algorithm was evaluated with different qualities of incident lighting. On a white monochromatic background, the μPAD array was tested under direct sunlight, shaded sunlight, diffuse fluorescent light, and smartphone flash lighting conditions. Each light source caused greater and consistent error for channels furthest from the reference point, particularly under shaded sunlight (maximum error = 11%), but the comparative overall error was low for all conditions (<5%) (Fig. 3d, top). The algorithm reduced this consistent error between the furthest channels as well as the global error, again proving useful for controlling measured signal differences from individual channel characteristics and overall lighting variation (Fig. 3d, bottom). However, these tests suggest that lighting quality does not as significantly influence imaging as do the height of the light source and the quality of the imaging background.

### Validation of Reagent-Based μPAD Assays for First-Order Contaminant Detection

The following reagent-based optical detection methods for our four water contaminants were adapted to our μPAD platform. Through initial calibration of our platform using single-target solutions, we first validated our platform’s quantitative performance as compared with that of the original assays.

### Chromium (VI) Assay Sensitivity and Dual Target Specificity Tests

**Figure 4:**
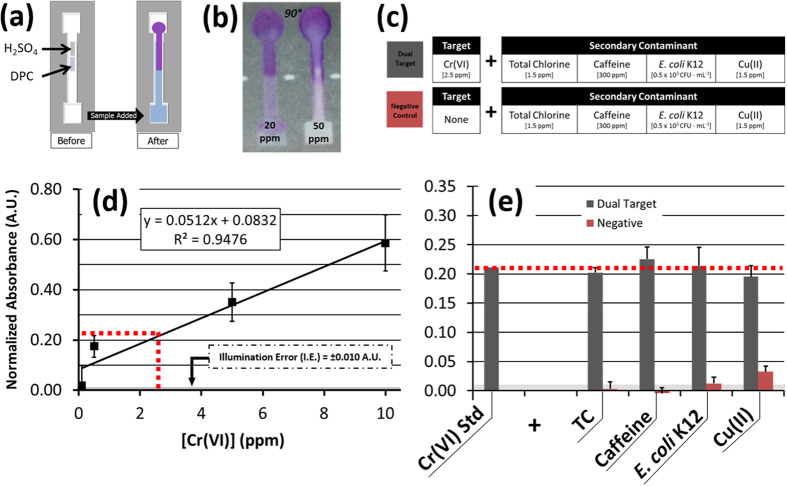
(**a**) A colorimetric μPAD assay detecting Cr(VI) using diphenylcarbazide (DPC) and H_2_SO_4_. (**b**) In the presence of increasing concentrations of Cr(VI), an increasing magenta color change is produced, as measureable by a change in normalized absorbance (A) of the green band (λ_max_ = 540 nm) reflected light intensity. (**c**) Dual-target and negative control solutions were assayed and compared with a (**d**) DPC-Cr(VI) standard curve created by single-target μPAD detection of Cr(VI) concentrations using DPC (n = 3). (**e**) There was no significant difference in normalized absorbance measured between the dual target control solutions and 2.5 ppm Cr(VI). Conversely, there was a significant difference between 2.5 ppm Cr(VI) and all negative control solutions (p < 0.05).

Cr(VI) concentrations between 0.05 ppm and 50 ppm were evaluated based on the change in absorbance of DPC (Fig. 4a,b)^27^. With our image processing and triple-reference normalization algorithm, the RGB green channel absorbance of DPC-Cr(VI) showed a significant and linear increase for increasing concentrations of Cr(VI) under ambient lighting conditions, and with a statistically significant dynamic range above the background illumination-based error for Cr(VI) concentrations from 1 ppm to 50 ppm (Supplementary Fig. S1). Based on this, a standard curve was derived for Cr(VI) concentrations between 0.1 and 10 ppm (Fig. 4d) and was used as a positive comparison for specificity tests with dual-target solutions (Fig. 4c). From this, the normalized absorbance of 2.5 ppm Cr(VI) was determined as A = 0.21 (Eq. 3). The same concentration of Cr(VI) maintained a statistically similar signal level when measured within separate dual-target mixtures, each with 1.5 ppm TC, 300 ppm caffeine, 0.5 × 10^3^ CFU ∙ mL^−1^ *E. coli* K12, and a separate heavy metal control of 1.5 ppm Cu(II) (p < 0.05, all) (Fig. 4e). In contrast, these same concentrations of secondary contaminants did not exhibit a statistically significant absorbance measurement when tested alone, demonstrating favorable specificity using our smartphone assay concept for Cr(VI) and its high-contrast complex with DPC (p < 0.05, all).

### Total Chlorine (TC) Assay Sensitivity and Dual-Target Specificity Tests

**Figure 5:**
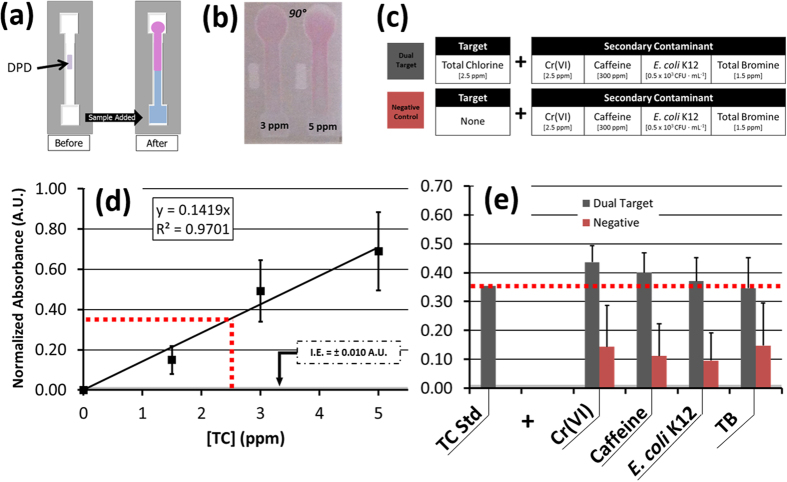
(**a**) A colorimetric μPAD assay for detecting total chlorine (TC) using N,N-diethyl-p-phenyldiamine sulfate (DPD). (**b**) With increasing TC concentration, an increasing lavender color change is produced, as measureable by a change in normalized absorbance of the green band (λ_max_ = 520 nm) reflected light intensity. (**c**) Dual-target and negative control solutions were assayed and compared with a (**d**) DPD-TC standard curve created by single-target μPAD detection of TC concentrations using DPC (n = 4). (**e**) There was no significant difference in normalized absorbance measured between the dual target control solutions and 2.5 ppm TC. Compared with the negative control solutions, there was only a significant difference between 2.5 ppm TC and *E. coli* K12 (p < 0.05).

TC concentrations between 1.5 ppm and 5 ppm were evaluated based on the change in absorbance they exhibited interacting with DPD (Fig. 5a,b)^28^. With the same image processing and normalization algorithm, the RGB green channel absorbance of DPD-TC increased linearly for increasing concentrations of TC under ambient lighting conditions, and with a dynamic range of 1.5 to 5 ppm as measured above the background illumination-based error (Fig. 5d). However, all absorbance measurements taken for TC concentrations below 1.5 ppm resulted in an unsteady signal, with limited statistical differentiation available between same order of magnitude concentrations.

This standard curve created for TC was used as a specificity test comparator. From this curve, an absorbance approximation of A = 0.36 (Eq. 3) was made for 2.5 ppm TC. Mixtures of a 2.5 ppm TC concentration were combined separately with 2.5 ppm Cr(VI), 300 ppm caffeine, and a 1.5 ppm TB (total bromine) inorganic ionic control species, and each exhibited a statistically equivalent absorbance, although with substantial variance (p < 0.05) (Fig. 5c,e).

However, in isolated negative control solutions the same concentrations of Cr(VI), TC, and TB could not be statistically differentiated from 2.5 ppm TC by absorbance measurements, demonstrating poor specificity of DPD (Fig. 5e). Only *E. coli* K12 saw a significant difference (p < 0.05). The unsteady signal seen for the single-target TC assays and this demonstrated lack of specificity may be due to the known instability of chlorine in solution, which is also known to cause challenges in the stability of target-agent detection complexes^32^. Such instability appears inherent to this assay, and thus would need to be conditioned by chemical changes to the assay rather than by optical methods.

### Caffeine Assay Sensitivity and Dual-Target Specificity Tests

**Figure 6:**
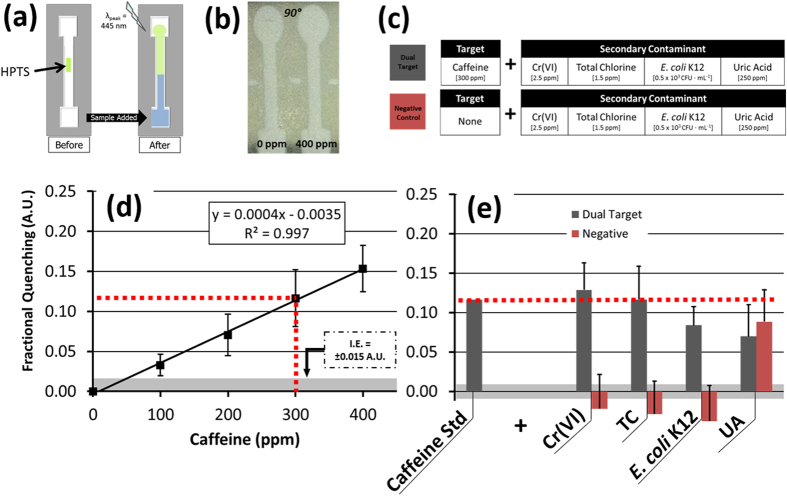
(**a**) A fluorescent μPAD assay for detecting caffeine using 8-hydroxypyrene-1,3,6-trisulfonic acid (HPTS). (**b**) Increasing caffeine concentrations in the presence of blue excitation wavelengths (λ_ex_ = 445 nm) produce a decreasing, or quenched, change in reflected green light intensity (λ_em, max_ = 512 nm). (**c**) Dual-target and negative control solutions were assayed and compared with a (**d**) HPTS-caffeine standard curve created by single-target μPAD detection of caffeine concentrations using HPTS (n = 4). (**e**) There was no significant difference in fractional quenching measured between the dual target control solutions and 300 ppm caffeine. However, the UA negative control solution also demonstrated no significant difference in quenching from the caffeine standard (p < 0.05).

Caffeine concentrations of 100 to 400 ppm were evaluated based on their exhibited change in green fluorescence quenching through their interaction with HPTS, as demonstrated initially by spectroscopy from an aqueous mixture (Fig. 6a,b)^29^. Using the μPAD, a similar quenching signal was isolated by the reflected intensity measured from the green channel of a smartphone’s RGB image. These images were captured using the smartphone’s white LED flash as a light source (λ_peak,blue_ = 445 nm). Following the image processing and normalization algorithm, the RGB green channel absorbance of HPTS-caffeine was found to increase linearly for increasing concentrations of caffeine under these conditions, as anticipated (Fig. 6d), with a 100 ppm limit of detection as measured above the background illumination-based error.

Following this single-target validation, the standard curve was used as a comparator for each dual-target and negative control solution (Fig. 6c). From this, a fractional quenching signal of Q = 0.12 (Eq. 4) was measured for 300 ppm caffeine. Mixtures of this same caffeine concentration combined separately with 2.5 ppm Cr(VI), 1.5 ppm TC, and a 250 ppm uric acid (UA) organic molecular control species each exhibited a statistically similar fractional quenching signal (p < 0.05) (Fig. 6e). Among the negative control comparison solutions, each target except for UA resulted in a fractional quenching signal that was significantly below that of the 300 ppm caffeine standard (p < 0.05). The UA solution was indistinguishable from the caffeine solution (Q = 0.09), which may be attributed to the chemical structure and functionality of the compound. Uric acid was selected as a comparator for HPTS specificity with caffeine testing partly because it is present in urine, as is caffeine following its consumption; however, the primary reason for its inclusion as a comparator was because of its functional similarity to caffeine. HPTS responds to the heterocyclic and chemical structure of caffeine, which is a purine compound^29^. Uric acid, a heterocyclic purine metabolic byproduct, might thus be expected to react similarly with HPTS, as was observed. Thus, although HPTS-based fluorescent detection of caffeine cannot be distinguished from detection of uric acid on this platform, we do see a nearly equivalent response to both compounds (Fig. 6e). As such, this test would not be able to verify the strict presence of caffeine, but smartphone-based μPAD detection can potentially be adapted to identify either component, which could be linked to urine excretions.

### *Escherichia coli* K12 Assay Sensitivity and Dual-Target Specificity Tests

**Figure 7:**
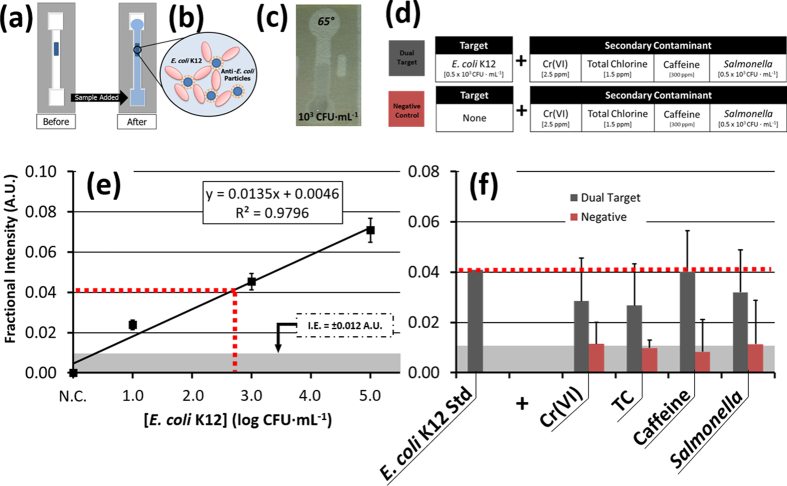
(**a**) A scattering-based μPAD assay for *E. coli* K12 via (**b**) immunoagglutination of polystyrene microparticles conjugated with anti-*E. coli* IgG. Through antibody-antigen binding, the presence of *E. coli* in a water sample leads to enhanced clustering of particle-*E. coli* complexes in the channel. (**c**) This change is optically detectable by smartphone imaging, and is correlated to *E. coli* K12 concentration by measuring the normalized change in Mie scattering intensity at 65° under ambient lighting conditions. (**d**) Dual-target and negative control solutions were assayed and compared with (**e**) single-target μPAD detection of *E. coli* K12 concentrations using anti-*E. coli* K12 IgG-conjugated microparticles as a scattering reagent (n = 4). (**f**) There was no significant difference in fractional quenching measured between the dual target control solutions and 0.5 × 10^3^ CFU∙mL^−1^ *E. coli* K12, whereas each negative control solution demonstrated a significant difference in intensity compared with the *E. coli* K12 standard (p < 0.05).

Water solutions containing *E. coli* K12 concentrations between 10^1^ and 10^5^ CFU∙mL^−1^ were assessed according to their interaction with anti-*E. coli* conjugated microparticles as these water samples flowed through the prepared paper channel (Fig. 7a,c). Our previous study has demonstrated that *E. coli* antigens are capable of traveling through this fabricated paper channel, and that as the water sample passes through the central microparticle-prepared channel segment these bacterial antigens facilitate the formation of microparticle clusters through attachments made with their antibody (Fig. 7b)^30^,^33^. The Mie-regime scatter intensity, measured from these clusters at 65° from the surface, is proportional to the concentration of *E. coli* K12 in solution and has thus allowed for quantification of these levels based on relative scatter, or fractional intensity measurements (Eq. 5). An initial standard curve was created for the normalized relative scattering from the microparticle clusters formed with *E. coli* K12 suspended in deionized water, and demonstrated a 10^1^ CFU∙mL^−1^ limit of detection as well as an excellent response and increase in scattering intensity for order of magnitude increases in the bacteria concentration (Fig. 7e).

Within that range, a sample of 0.5 × 10^3^ CFU∙mL^−1^ *E. coli* K12 was combined in separate dual target mixtures with 1.5 ppm TC, 300 ppm caffeine, and 0.5 × 10^3^ CFU∙mL^−1^ *Salmonella* Typhimurium, which served as a biological control target (Fig. 7d). The assay demonstrated a similar, although lower, increase in relative intensity as compared with the isolated *E. coli* K12 solution (p < 0.05) (Fig. 7f). The scattering intensity was attenuated most in the mixtures containing Cr(VI) and TC, which served to break up the microparticle-*E. coli* K12 clusters (Supplementary Fig. S2).

This may be caused by destruction of viable *E. coli* colonies and denaturation of their free antigens and/or the antibodies. In these instances, although the bacteria may not be viable their presence is still detectable, though not necessarily reproducibly quantifiable. This is because the precise distribution of free antigens may vary from solution to solution for the same starting concentration of viable *E. coli* K12, especially within the diffusion-limited paper fibers. However, further studies would be needed to verify the extent of these interactions.

## Conclusions

This method of smartphone image filtering and triple-reference normalization advances the reproducibility of smartphone-quantified μPAD assays for mobile water quality monitoring. Particularly for stable reagent-based assays, our technique may be a valuable mechanism toward continuous quantitative measurement of optically detectable μPAD assays under varying environmental conditions. This algorithm significantly reduces the impact of primary error sources, such as major lighting variation patterns and lighting quality changes between assays. Additionally, it helps to eliminate intra-channel error due to fine variations in μPAD surface features and saturation conditons. However, this algorithm is limited in its ability to completely remove partial μPAD shadowing, non-uniformity in imaging background reflection, and varied imaging height between compared assays. When used for testing species which are known to be unstable, such as *E. coli* K12 in the presence of toxic secondary contaminants, additional stabilization methods or reagents may be necessary to permit signal reproducibility. With accommodations made to prevent these sources of error, though, the algorithm affords order-of-magnitude quantification and specificity of detection for the assays under variable levels of illumination, providing one advantageous route for achieving the consistent quantification needed for field-ready smartphone-integrated μPAD assays without relying on secondary smartphone attachments.

## Methods

### Standard Solutions for Target Water Contaminants and Assay Reagents

Separate standard solutions for each of the tested water quality parameters were made in deionized water from a Milli-Q water purification system (18.2 MΩ∙cm; Milford, MA, USA). Deionized water was selected as the primary diluent in an effort to mitigate sources of error external to the environmental conditions (e.g. different lighting, imaging, and paper surface conditions) under evaluation in this work. Serial dilutions of Cr(VI) concentrations between 0.05 and 50 ppm were created from a stock of 1 M CrO_3_ (Sigma-Aldrich, St. Louis, MO, USA). Total chlorine (TC) concentrations between 0.5 and 5 ppm were serially diluted from a stock of 15 ppm NaClO (Sigma-Aldrich) prior to each test in chlorine demand-free glassware. Caffeine concentrations between 0.5 and 10 mM were diluted from a stock of 50 mM caffeine (Sigma-Aldrich). For the representative biological contaminant, lyophilized *Escherichia coli* K12 (Sigma-Aldrich) was cultured in Lysogeny Broth (LB; Remel, Lenexa, KS, USA) for 6–8 hours to a concentration of 10^8^ CFU∙mL^−1^, which was validated through a Lysogeny broth (LB) agar (BioExpress, Kaysville, UT, USA) plate counting method and subsequent correlation of the *E. coli* K12 solution absorbance at 600 nm. This stock was separated from the LB broth via centrifugation, resuspended in 0.2 mM PBS (pH 7.2 ± 0.2), and serially diluted to assay concentrations between 10^1^ and 10^5^ CFU∙mL^−1^.

### μPAD Design and Fabrication

The μPAD assay platforms were fabricated through a negative-resist photolithography process described in our previous work^30^. The final channels were composed of cellulose chromatography paper (pore size = 10–20 μm; thickness = 100 μm; GE Healthcare, Maidstone, Kent, UK) bordered by SU-8 2010 polymer (Microchem, Newton, MA, USA) that was cross-linked through ultra-violet radiation, creating a hydrophobic barrier around the perimeter of each hydrophilic channel. Each channel (3 mm wide × 11.5 mm long) was fabricated with a sample loading pad (4.5 mm wide × 3.5 mm long) at the inlet and an absorbent pad (5.5 mm wide × 6.5 mm long) at the outlet. One additional rectangle was patterned adjacent to but separate from the channel center to allow for comparison of the channel illumination compared with the peak “white” illumination available to the paper channel without target, reagent, or water interference.

### Reagent-Based μPAD Assay Imaging

Prior to each experiment, the μPAD was fixed horizontally at 16 cm from the lens of the smartphone (iPhone 4S; Apple, Cupertino, CA, USA), which was fastened into a precisely controlled angular rotation stage for initial experiments (Thorlabs, Newton, NJ, USA). For single-target assays, the auto-exposure and auto-focus were locked on the central paper channel surface at 90° for Cr(VI), TC, and caffeine assays and at 65° for *E. coli* K12 assays – the optimum scattering detection angle determined through previous work (Fig. 1d)^30^. For dual-target assays using *E. coli* K12, channels containing Cr(VI), TC, and caffeine were also imaged at 65°, which allowed similar though slightly reduced levels of quantification compared with 90° (Supplementary Fig. S3).

Two images were collected for each μPAD assay: the first image after loading and drying a 2 μL sample of the target-specific reagent solution to the center of the channel, and the second image after loading an 8 μL sample of the target water sample to the channel inlet and allowing it to flow completely through the channel for 10 minutes following the addition of Cr(VI), TC, and caffeine samples to the channel, or 30 seconds following *E. coli* K12 samples.

### Image-Processing Algorithm and Bias Correction

Ambient lighting was provided by a typical fluorescent bulb (λ_peak,green_ = 544 nm) for absorbance and relative intensity measurements, and by a smartphone LED flash (λ_peak,blue_ = 445 nm) for fluorescent quenching measurements. Each image was initially processed through ImageJ (National Institutes of Health, Bethesda, VA, USA) using a fast-Fourier transform (FFT) based digital bandpass filter (available in ImageJ) in order to provide illumination bias correction. The FFT bandpass filter was applied with 1% directional tolerance to large structures down to 30 pixels, and to small structures below 3 pixels.

Next, the mean pixel intensity from each detection region (I_s_, I_b_, I_p_, and I_w_; pixels n > 100 each) was measured from the green channel portion of the smartphone RGB image, as separated through ImageJ. In the first image, the mean intensity of the channel center was measured where the reagents were loaded and dried, and served as a background intensity (I_b_) reference (Fig. 1b). The second image was collected 10 minutes after Cr(VI), TC, and caffeine samples were added to the channel, or 30 seconds after *E. coli* K12 samples were added (Fig. 1c). In both cases, the channels were still wet. In this image, the mean intensity of the channel center point was measured, and provided the target-reagent signal intensity (I_s_). Two additional reference points were then taken from the same image. A peak white reference (I_p_) point was taken from a dry rectangle patterned adjacent to the channel center, identifying the maximum illumination available to the dry paper channel. A wetted reference (I_w_) point was also taken at the sample loading inlet, which allowed calibration for moisture-induced coloration differences between channels.

The typical order of these reflected intensities was, for the case of absorbance and quenching, 255 > I_p_ (the peak illumination of dry paper) >I_b_ (the dried reagents in the channel) >I_w_ (the wetted paper without target-reagent interactions) >I_s_ (the target-reagent signal from the wetted channel) >0. For the case of relative (scattering) intensity measurements, only the typical I_w_ and I_s_ signal magnitudes were swapped since the scattered intensity signal was higher in the presence of the target. Therefore, the wetted target-reagent signal can be normalized as I_s_/I_w_ (Eq. 1), which accounts for the target-reagent signal change as a fraction of the signal change due to moisture in the channel. The dry reference is normalized as I_b_/I_p_ (Eq. 1), which accounts for the maximum illumination available to the reagent in the dry channel as a fraction of the maximum total illumination available to the dry paper within the given lighting conditions. These reference intensity values were incorporated into the above-mentioned triple-reference point normalization algorithm (Eq. 1 and Eq. 2).

### Algorithm Fitness Testing

The FFT digital bandpass filter and triple reference normalization scheme were tested for their performance in handling changes in the lighting field (lighting angle ϕ; lighting source/quality) and changes in the imaging field (height of smartphone; background absorbance/reflectance characteristics). In each test, nine adjacent channels on a single μPAD were fixed within the smartphone imaging field and the smartphone white-balance and focus were locked on the detection region of the central channel (channel 5), denoted as the focal center (FC). For each set of tested conditions, images were collected and evaluated before and after adding 8 μL of DI water to each channel. Fractional intensity measurements were taken for each channel (R, Eq. 5) and were compared with that of the FC channel as a measure of error between them.

The first fitness test evaluated the response of this algorithm under directional lighting conditions. The nine channel μPAD array was positioned horizontally parallel to the smartphone camera at a distance of 16 cm and was then revolved and imaged around a fluorescent point light source and at angular intervals of ϕ = 45° intervals along a circumferential path (radius = 92 cm) over a black background. During rotation, the μPAD orientation was maintained such that when ϕ = 0° the channels were illuminated from the direction of their inlet, and when ϕ = 180° they were illuminated from the direction of their outlet.

The second fitness test evaluated the response of this algorithm under various imaging heights. The μPAD array was fixed within the center of the imaging field over a black background and was imaged from heights of 9.2 cm, 13.0 cm, 16.8 cm, and 20.3 cm.

The third fitness test evaluated the response of the algorithm for channels assayed over opposite imaging backgrounds. The μPAD array was imaged from a fixed height of 16 cm and was assayed using a black and a white background separately.

The fourth fitness test evaluated the response of the algorithm for channels assayed under different lighting quality conditions. The μPAD array was imaged from a fixed height of 16 cm over a white background under full sunlight, shaded sunlight, fluorescent laboratory light, and smartphone flash light.

### Cr(VI) Assay

Our Cr(VI) detection method was adapted from the EPA Method 7196A, in which a high-contrast magenta color change is produced by 1,5-diphenylcarbazide (DPC; Sigma-Aldrich) in response to a proportional concentration of Cr(VI)^27^. This magenta color is verified by quantifying the aqueous solution’s green absorbance at 540 nm using a spectrometer. In its adaptation to our μPAD platform, the lower-center of each channel was loaded with 2 μL of 5 M DPC prepared in acetone, while the upper-center was loaded with 1 μL of 1 M H_2_SO_4_. After preparation, drying, and imaging of the channel background, Cr(VI) solutions were added to separate channels and were allowed 10 minutes for flow and color development, which was quantified through normalized absorbance (Eq. 3 and Fig. 4a,b).

### Total Chlorine (TC) Assay

TC detection was adapted from the EPA-accepted Thermo Orion Method AC4P7, in which a medium-contrast lavender color change is produced by N,N-diethyl-p-phenyldiamine sulfate (DPD; Thermo Fisher, Waltham, MA, USA) in response to a proportional concentration of chlorine species^28^. This color change was initially verified by quantifying the aqueous solution’s green absorbance at 520 nm through spectroscopy. For detecting TC on paper, the center of each channel was loaded with 2 μL of 50% DPD. Following channel preparation and background imaging, TC solutions were added to separate channels. For smartphone-integrated detection, the stable color change was imaged after 10 minutes and was quantified under ambient lighting using the same normalized absorbance relationship (Eq. 3 and Fig. 5a,b).

### Caffeine Assay

Detection of caffeine was adapted from a method described by Rochat *et al*.^29^. In this, the low-contrast green fluorescent quenching of 8-hydroxypyrene-1,3,6-trisulfonic acid (HPTS; Sigma-Aldrich) in response to caffeine is quantified by the aqueous solution’s quenching at 512 nm (470 nm excitation) as measured by spectroscopy. For smartphone-integrated detection, the HPTS-caffeine complex was imaged using the smartphone’s white LED flash (λ_max,blue_ = 445 nm) and the green channel intensities were quantified using the defined fractional quenching relationship (Eq. 4 and Fig. 6a,b).

### *E. coli* K12 Assay

Detection of *E. coli* K12 was adapted from the previous work based on the change in Mie scattering of immunoagglutinated microparticles^30^,^33^. In this, anti-*E. coli* conjugated microparticles bind to *E. coli*, forming larger clusters that cause low-contrast but measureable changes in detectable scattering at defined angles from the surface (Fig. 7a–c).

In preparation for *E. coli* K12 detection using the μPAD platform, each channel was prepared with 2 μL of prepared 0.91 μm diameter carboxylated polystyrene microparticles (Magsphere, Pasadena, CA, USA) conjugated with goat polyclonal antibody to *E. coli* (47385G, Meridian Life Sciences, ME, USA) to a final concentration of 0.015–0.030% (v/v) particles, according to our previous study^30^. Following channel preparation and background imaging, *E. coli* K12 solutions were prepared with a final concentration of 10% v/v of 0.04% Tween 20 in order to release antigens from *E. coli* colonies. The *E. coli* K12 were added to the channels and flowed for 30 seconds, after which the second image was taken. *E. coli* K12 concentration was quantified according to the normalized fractional (scatter) intensity from the surface, as defined above (Eq. 5).

### Dual-Target μPAD Assays for Specificity Analyses of Second-Order Contaminant Interactions

Following validation of the single-target μPAD assays, mixed-target solutions were created to test the specificity of smartphone detection for each paper-based assay amid second-order contaminant interactions. Each of the individual water contaminants was mixed separately with each of the other three water contaminants (e.g., TC, caffeine, and *E. coli* K12 for the Cr(VI) assay), and one additional control solution was created with an additional contaminant bearing functional similarity to the original contaminant. For Cr(VI), a functional control of 3 ppm Cu(II) from an aqueous suspension of CuSO_4_ (Sigma-Aldrich) was used as such functionally similar contaminant. For TC, a 1.5 ppm solution of aqueous tetraoctylammonium bromide was used as a test for total bromine (TB) (Sigma-Aldrich). For caffeine, a 300 ppm solution of uric acid was used, a purine-originated compound found in urine, thus bearing potentially similar anthropogenic origins with caffeine. For *E. coli* K12, a 0.5 × 10^3^ CFU∙mL^−1^ solution of *Salmonella* Typhimurium was used as a negative control comparison. Each of these solutions was then tested with the reagent specific to the primary target and data was collected for each according to the same process used for single-target assays. A series of negative control assays were tested simultaneously using the same concentrations of the isolated secondary contaminants for specificity comparison. A standard curve was also simultaneously created for each primary contaminant according to the same process used for initial assay validation. The signal from each dual contaminant mixture and secondary-contaminant negative control mixture was compared with that of the equivalent concentration signal from the standard curve. This provided a test of the response exhibited by each reagent in the mixed-solution both in the presence and in the absence of its primary target.

### Statistical Comparison between Assays

The error in intensity measurements between channels within a single image were evaluated through percent error (Δ) and standard error measurements. Pixel samples of each representative channel region (n > 25) were measured for their mean intensity and standard error, which was represented as a fraction of the same mean intensity standard error value measured for the equivalent region of the reference channel.

Between concentration levels of each single target assay and between separate μPAD trials of each deionized water control assay, significance measurements were made through the two-sample unpaired t-test. Between dual-target assays, negative control assays, and their equivalent single-target assay comparisons, significance measurements were made through ANOVA.

## Additional Information

**How to cite this article**: McCracken, K. E. *et al*. Multimodal Imaging and Lighting Bias Correction for Improved µPAD-based Water Quality Monitoring via Smartphones. *Sci. Rep.* **6**, 27529; doi: 10.1038/srep27529 (2016).

## Supplementary Material

Supplementary Information
