# Supplementary material for: Multimodal Imaging and Lighting Bias Correction for Improved μPAD-based Water Quality Monitoring via Smartphones
Source: Sci Rep. 2016 Jun 10;6:27529. doi: 10.1038/srep27529 (PMC4901345; doi:10.1038/srep27529)
Supplement: Supplementary Information [file srep27529-s1.pdf]

Multimodal Imaging and Lighting Bias Correction for Improved µPAD-based Water Quality Monitoring via Smartphones

SUPPLEMENTARY INFORMATION

Katherine E. McCracken,[a] Scott V. Angus,[a,†] Kelly A. Reynolds,[b,*] Jeong-Yeol Yoon[a,*]
[a]Department of Agricultural & Biosystems Engineering and [b]Mel and Enid Zuckerman College of Public Health, The University of Arizona, Tucson, Arizona 85721, United States

[*]Corresponding authors. E-mail addresses: reynolds@email.arizona.edu (K.A. Reynolds) and jyyoon@email.arizona.edu (J.-Y. Yoon).
[†]Current affiliation: Guild BioSciences, a business unit of Guild Associates Inc., Dublin, Ohio 43017, United States

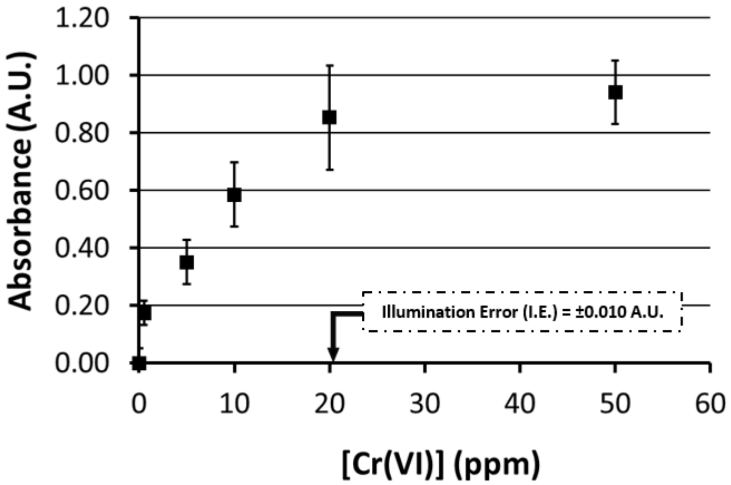

Supplementary Figure S1. (a) A single-target µPAD assay using DPC as a colorimetric reagent for quantifying Cr(VI) concentrations between 0.5 and 50 ppm based on absorbance was conducted (n = 5). This was conducted in order to verify that our paper-based assay adaptation functioned similarly to the original spectroscopic assay, the EPA Method 7196A. Our functional assay range was determined to be 0.5 ppm to 20 ppm Cr(VI). Concentrations above 0.5 ppm were distinguishable from background illumination-based error, and high concentrations above 20 ppm were not significantly distinguishable from one another.

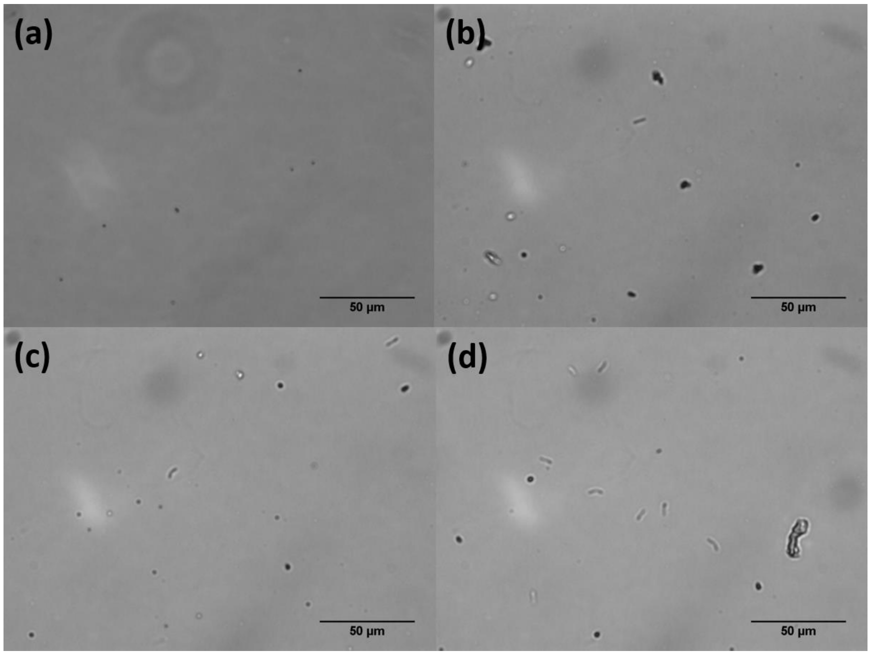

Supplementary Figure S2. (a) Comparative anti-*E. coli* microparticle flocculation in deionized water versus (b) immunoagglutination in the presence of 10^5 CFU·mL^{-1}. (c) Antibody-antigen binding between IgG particles and bacteria is compromised in the presence of 2.5 ppm Cr(VI) and (d) 1.5 ppm TC, as evidenced by a larger percentage of free, unbound bacteria as well as smaller interparticle clusters.

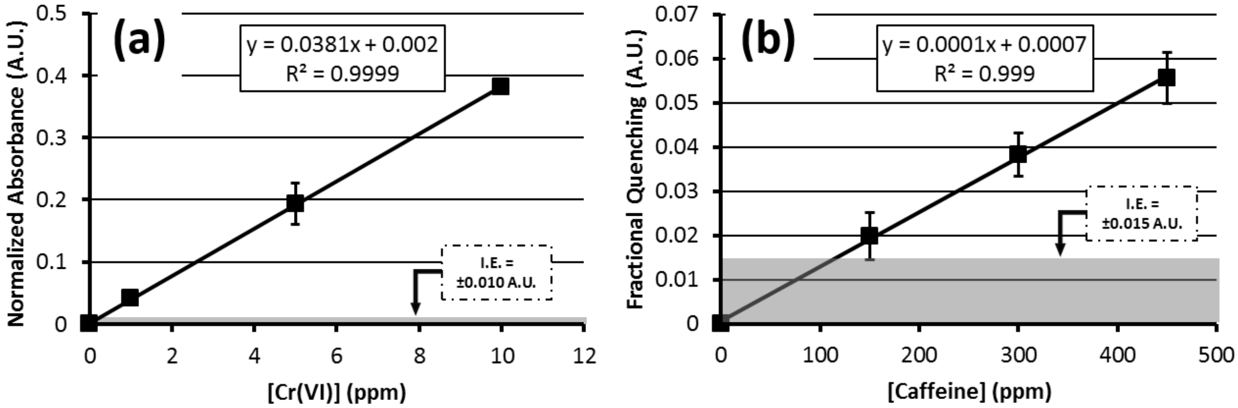

Supplementary Figure S3. Absorbance (a, for Cr(VI) using DPC) and quenching (b, for caffeine using HPTS) measurements (n = 3) of single-target µPAD assays from the smartphone images taken at 65° (the angle used for *E. coli* assay) in comparison to 90° used to generate the data shown in Figs. 4-7 (n = 3).
